# Examining the Validity of the Phonon Gas Model in Amorphous Materials

**DOI:** 10.1038/srep37675

**Published:** 2016-12-05

**Authors:** Wei Lv, Asegun Henry

**Affiliations:** 1 George W. Woodruff School of Mechanical Engineering, Georgia Institute of Technology, Atlanta, GA 30332, USA; 2 School of Materials Science and Engineering, Georgia Institute of Technology, Atlanta, GA, 30332, USA

## Abstract

The idea of treating phonon transport as equivalent to transport through a gas of particles is termed the phonon gas model (PGM), and it has been used almost ubiquitously to try and understand heat conduction in all solids. However, most of the modes in disordered materials do not propagate and thus may contribute to heat conduction in a fundamentally different way than is described by the PGM. From a practical perspective, the problem with trying to apply the PGM to amorphous materials is the fact that one cannot rigorously define the phonon velocities for non-propagating modes, since there is no periodicity. Here, we tested the validity of the PGM for amorphous materials by assuming the PGM is applicable, and then, using a combination of lattice dynamics, molecular dynamics (MD) and experimental thermal conductivity data, we back-calculated the phonon velocities for the vibrational modes. The results of this approach show that if the PGM was valid, a large number of the mid and high frequency modes would have to have either imaginary or extremely high velocities to reproduce the experimental thermal conductivity data. Furthermore, the results of MD based relaxation time calculations suggest that in amorphous materials there is little, if any, connection between relaxation times and thermal conductivity. This then strongly suggests that the PGM is inapplicable to amorphous solids.

There is a gap in the theoretical understanding of heat conduction through amorphous solids and also a lack of numerical methodologies for predicting the associated mode level thermal conductivity contributions. Understanding the contributions to thermal conductivity from different phonons is important, because once the dominant phonons and their transport mechanism can be understood, a means by which their contributions can be manipulated can then be explored^1^,^2^,^3^,^4^,^5^,^6^,^7^,^8^. For example, if the thermal conductivity of amorphous silica (a-SiO_2_) could somehow be reduced by an order of magnitude it might enable fabrication of lower thermal conductivity insulation or windows, which could have a significant impact on the space heating/cooling requirements for homes and industries. Towards this end we seek a robust theoretical foundation from which we can understand heat conduction in amorphous materials.

Essentially all understanding of phonon transport is based on the phonon gas model (PGM) whereby phonons are treated as quasi-particles and their transport is subsequently modeled like that of gas molecules that carry energy 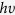 (instead of kinetic energy 1/2 *mv*^2^, since phonons are massless). The PGM then ascribes every phonon with the following contribution to the heat flux,


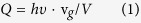


where *V* is the volume per mode, v_*g*_ is phonon group velocity, and 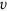 is the phonon frequency. Equation (1) expresses the essence of the PGM and it is at the heart of virtually every expression for phonon transport, because the derivation of almost all expressions begin with such a statement^9^,^10^. This, however, becomes problematic for materials that do not have a well-defined phonon dispersion and therefore do not have well-defined velocities. The central problem is the fact that the phonon velocity is a critical variable in Eq. (1) since it determines the rate at which its energy moves through the material (i.e., from a region of higher temperature to lower temperature).

In reality all phonons share two properties, which is that of a well-defined frequency and time scale over which their amplitude remains correlated (e.g., a relaxation time), since they are most generally/mathematically just quantum oscillators. Wavelengths, wave vectors, velocities and mean free paths (MFPs), on the other hand, are not general properties of normal modes of vibration, because they become ill-defined in when there is disorder^11^. Despite this lack of generality, the PGM has been used almost ubiquitously across all material classes^5^,^10^,^12^,^13^,^14^,^15^,^16^,^17^. As a result, the notion that every phonon has a MFP and that the MFP is the primary descriptor that makes one material better at conducting heat than others has pervaded almost all phonon transport literature^5^,^7^,^18^,^19^,^20^,^21^,^22^.

From the PGM perspective, thermal conductivity depends on the individual mode heat capacities (*c*), phonon group velocities (v_*g*_), and relaxation times (*τ*) as,


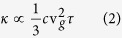


Considering the fact that the thermal conductivity of solids spans about 5 orders of magnitude (0.1–10,000 Wm^−1^K^−1^), it is instructive to examine which variables in Eq. (2) must be responsible for the range of κ observed in nature. For example, the heat capacities are essentially determined by the phonon density of states and the velocities scale with the speed of sound. The heat capacity from phonons in virtually all materials reaches the same maximum value at high temperatures, which is 3*k*_*B*_ per atom. Similarly, the speed of sound in solids is generally in the range of 1,000–10,000 m/s. However the relaxation time of phonons in solids could span more than three orders of magnitudes. Hence the PGM asserts that the relaxation times that are an important descriptor for explaining why transport is inhibited or more facile in different materials. Furthermore, the temperature dependence of thermal conductivity in a given class of materials is largely determined by the temperature dependence of the relaxation times^23^,^24^. Given this rather general theoretical framework imparted as a natural consequence^9^,^10^ of taking Eq. (1) to be true, and the fact that the relaxation times are predominantly responsible for the temperature dependence of thermal conductivity above cryogenic temperatures, one can devise a scheme by which to assess the validity of the PGM.

The validity of the PGM becomes questionable for a class of materials such as amorphous materials, due to the inability to define the phonon velocities. Here it is important to emphasize that periodicity is an inherent requirement for rigorously defining the phonon velocities, since it requires that one define the phonon wave vectors, which in turn require periodicity. Therefore in an amorphous material, where there is no long-range periodicity, it is useful to assess whether or not one can still utilize the PGM framework in order to make sense of the thermal conductivity. If for example, one could still rationalize the behavior of amorphous materials with the PGM, then one can think of somehow defining an effective MFP for different modes and continue with the general viewpoint that has been used for almost all other materials. However, if after such an assessment one determines that the PGM is inapplicable, then one must then proceed to consider alternative descriptions of the heat flow and a revised physical picture for the transport.

To assess if one can rationalize the PGM for a given class of materials one could use a combination of experimental data for thermal conductivity (κ) and atomistic level calculations of the mode heat capacities (*c*) and relaxation times (*τ*) to then back-calculate the corresponding velocities that would have to be ascribed to each mode in order to reproduce the experimental thermal conductivity. Here, the idea that one can back-calculate the velocities, is based on the basic assertion that κ, *c*, and *τ* together contain all of the temperature dependence, and any temperature dependence associated with v is negligible.

No matter how one tries to rationalize the velocities of phonons, it is difficult to imagine that somehow the velocities would not scale with the elastic modulus (e.g., the bulk and shear modulus) or at least be limited to values on the same order of magnitude as the speed of sound (e.g., assuming they are not polaritons^25^,^26^). Furthermore, one would also expect that any temperature dependence associated with the velocities of modes in a given material must still exhibit similar temperature dependence to the speed of sound or modulus, which generally do not show strong temperature dependence for amorphous materials^27^. For example, in the case of amorphous silicon (a-Si)^28^ and a-SiO_2_^29^,^30^, the change in modulus with temperature is less than 10% for both a-Si between 200–800 K and a-SiO_2_ between 100–1200 K. Consequently, the sound velocity, which is proportional to the square root of the modulus, then only changes by less than 4% over these respective temperature ranges. Hence an important, yet physically well-reasoned assumption herein is that phonon velocities (v_*g*_(*n*)), whether defined or ill defined, must still exhibit negligible temperature dependence (e.g.,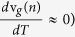 as compared to the heat capacity and relaxation times. This logic then yields a framework whereby one can use a combination of experimental data and relaxation times derived from lattice dynamics and molecular dynamics (MD) to assess if the resulting calculation for the phonon velocities is sensible. Towards this end, we calculated the mode relaxation times from normal modal analysis (NMA)^2^,^12^,^22^ using the Tersoff potential^31^,^32^ for amorphous silicon (a-Si) and amorphous silica (a-SiO_2_)^33^,^34^. We calculated individual mode relaxation times at six different temperatures (30 K, 100 K, 200 K, 400 K, 800 K, 1200 K) for a-SiO_2_. Then we simply interpolated between the data obtained at each temperature to yield a continuous function for each mode’s relaxation time as a function of temperature from 30 K to 1200 K. It is well appreciated that relaxation time results derived from classical MD are incorrect at low temperatures. However, based on Turney, McGaughey and Amon’s work^35^, it appears reasonable to expect that the classical MD relaxation times are within an order of magnitude and should generally exhibit the appropriate scaling with temperature, which, as will be shown later, is most critical to the subsequent conclusions. Therefore, assuming the PGM is valid, we then calculated the velocity squared via, v_*g*_(*n*)^2^ = 3*κ*(*T*)/[*c*(*T*, *n*)*τ*(*T*, *n*)] for each mode.

Conceptually, one could argue that there does not exist a unique solution for 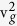, but in practice there is. Since one cannot rationalize the velocities having strong temperature dependence, they are constant and therefore unique for every frequency. As a result, one can simply begin by solving for the velocities at low temperatures where the majority of the modes’ heat capacities are effectively zero. In this temperature range one can easily find a unique solution to 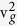, because only a small portion of the low frequency modes can contribute to the thermal conductivity. Once
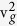
for low frequency modes is determined, one can then gradually progress to successively higher temperatures, solving for a unique function 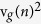
at successively higher and higher frequencies with mode relaxation time *τ*(*T*, *n*) and specific heat *c*(*T*, *n*) at this temperature. The detailed calculation procedures are described in the supplementary materials.

The calculated mode-wise squared velocity v*g*(*n*)^2^ and the magnitude of the real and imaginary part of the velocity (e.g. since now the phonon velocity can become imaginary if 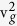 is negative) is shown in Fig 1. Note that in Fig. 1, 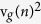
is normalized by the longitudinal velocity (6500 m/s) calculated from bulk modulus^36^. From Fig. 1(a) it is apparent that v*g*(*n*)^2^ is negative for many mid-frequency modes. Hence as shown in Fig. 1(b), there are a great number of modes that would have to have imaginary group velocities in order to reproduce the experimental data. The most significant contributor to this requirement is the rise and then sharp drop in relaxation times between 20–30 THz (see Fig. 2d). This feature in the relaxation times requires imaginary velocities, if these phonons’ contributions to the thermal conductivity are proportional to their relaxation times. Furthermore, Fig. 1(b) shows that the magnitude of v*g*(*n*) is unreasonably large (>100X the speed of sound) for some middle and high frequency modes. Neither of these two results are sensible or can be rationalized. Imaginary velocities have no sensible interpretation in the PGM and velocities that are orders of magnitude greater than the speed of sound are nonsensical, even if one assumes the real quantum relaxation times are an order of magnitude larger. These two observations then lead us to conclude that one cannot self-consistently rationalize the usage of the PGM in a-SiO2 and it is likely that the lack of applicability may extend to many, if not all, amorphous materials.

To understand the thermal conductivity of amorphous materials more properly, we consider an alternative view that can rationalize the observed behaviors in amorphous materials in a self-consistent framework, namely that of the recently developed Green-Kubo modal analysis (GKMA) method by Lv and Henry^33^. GKMA leverages the fact that the Green-Kubo method, which has been extensively used in molecular dynamics simulations^2^,^37^,^38^,^39^,^40^,^41^, is valid for any phase of matter as long as the response to a thermal perturbation remains in the linear regime, which is likely the case for almost all terrestrial scenarios and technologically relevant situations. The details of the GKMA formalism are presented elsewhere^33^, but in summary, GKMA combines the Green-Kubo (GK) and lattice dynamics (LD) formalisms to write the thermal conductivity as a direct summation over modal contributions, derived from a direct modal decomposition of the heat flux operator as shown in the supplementary materials, ref. 42. The result is then an expression for thermal conductivity that yields each individual mode’s contribution, without having to define the mode’s velocity or invoke an expression based on the PGM.

Previous work by Lv and Henry has shown that GKMA yields excellent agreement with experiments on the thermal conductivity of amorphous materials, especially a-Si and a-SiO_2_^33^,^34^. Thus its results can be regarded as not only accurate, but also meaningful towards deriving improved insight and assessing the validity of the PGM. However, one could still argue that even though GKMA provides a fundamentally different physical picture for the transport (e.g., based on correlation and not scattering^34^,^43^), that somehow one could possibly still view the problem from a PGM point of view, via some effective MFP treatment. Here, we examine this question more deeply and show that the behavior in amorphous materials is distinctly different from what can be rationalized by the PGM and thus the PGM is a fundamentally problematic way of viewing phonon transport in amorphous materials, since one cannot rationalize the usage of a MFP based explanation.

In this study we used the same atomic structure of a-Si and a-SiO_2_ as previous work^33^,^34^ and all of the same simulation procedures have been employed here. All the MD simulations are conducted in Large-scale Atomic/Molecular Massively Parallel Simulator (LAMMPS)^44^ and lattice dynamics calculations were performed using the General Utility Lattice Program (GULP)^45^. In Fig. 2(a) and (b), the thermal conductivity accumulations with respect to frequency *κ*(*ω*) are shown at different temperatures. The accumulation here is directly calculated from GKMA without a quantum specific heat correction. All modes are excited in MD and therefore they all have identical heat capacity 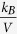, due to the classical nature of MD. As a result, classical GKMA results do not incorporate the temperature dependence of the heat capacity. After dividing by the constant heat capacity 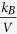 for every mode, one can then think of the accumulation as a thermal diffusivity accumulation, where 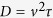. If one were to then try and rationalize the results in terms of the PGM, one would expect that the corresponding thermal diffusivity contributions must follow the same temperature dependence as the relaxation times determined from MD.

In both a-Si and a-SiO_2_, the relaxation times drop significantly as temperature increases. However the thermal conductivity contributions shown in Fig. 2(c) and (d) do not decrease in the same way. From the relaxation time *τ*(*ω*) and thermal conductivity accumulation *κ*(*ω*) in a-Si and a-SiO_2_, the trend required for consistency with the PGM is not observed. In a-SiO_2_, for example, above 400 K, the thermal conductivity accumulations are approximately identical, even though the relaxation times decreased by about a factor of two. Furthermore, at 400 K, 800 K and 1200 K, there is no clear reduction of thermal conductivity, and it should be emphasized that the temperature dependent thermal conductivity obtained in these simulations is in excellent agreement with experiments, once quantum corrected. This further suggests that the thermal conductivity accumulations are both accurate and meaningful.

From Fig. 2(d), it is also clear that the effects of anharmonicity do not seem to alter the thermal conductivity contributions between 400–800 K for a-SiO_2_. However, at 100 K the accumulations are very different from others, which shows that, how anharmonicity affects the thermal conductivity contributions at low temperatures is different than at high temperatures. As for a-Si, comparing 300 K and 800 K, the relaxation times clearly decrease (Fig. 2(a)), yet somehow the thermal diffusivities increase (Fig. 2(c)), which again contradicts the expected behavior based on the PGM.

From the results in Fig. 2, one can conclude that thermal diffusivity in amorphous materials does not generally exhibit direct correspondence with the relaxation times. Therefore the idea that all phonon contributions to thermal conductivity can be thought of in terms of relaxation times seems problematic. For modes that do not behave like propagating modes, their contributions to thermal conductivity appear to exhibit almost no connection to their relaxation times and it is desirable to somehow assess this quantitatively. Directly examining the 
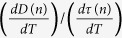
, where *D*(*n*) is the mode diffusivity leads to values that oscillate about zero, because in GKMA the thermal conductivity is a summation of many positive and negative thermal conductivity contributions from individual modes. Since the mode heat capacity is always positive, the individual mode diffusivities tend to fluctuate around zero in the GKMA approach, since the correlation functions yield both negative and positive values that ultimately sum to a net positive transport coefficient^33^. Therefore to better quantitatively assess the level of disconnect between the rather consistently decreasing relaxation times, and the often constant and sometimes increasing mode diffusivities, instead of examining the ratio, 
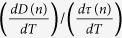
, we examine its accumulation,


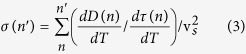


whereby we are primarily interested in the sign of this accumulation as opposed to its actual value. In essence, *σ* helps us to assess whether or not the diffusivities and relaxation times are increasing or decreasing together or whether or not they have opposing temperature trends more often than not. By analyzing the accumulation, instead of the argument in the sum, one can discern the net effect of adding many modes together from the individual mode ratios, which oscillate about zero.

In Eq. 3, v_*s*_ is the (maximum) sound velocity calculated from the bulk modulus calculated from GULP^45^, which is used to non-dimensionalize *σ*. For c-Si we take v_*s*_ = 9,000 m/s, and for a-Si the speed of sound is taken as 7,909 m/s. Since we do not have a continuous function for 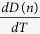 and 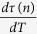 with respect to temperature, we used Δ*D*/Δ*T* and Δ*τ*/Δ*T* between the temperatures at which GKMA results were computed. This ratio represents the slope in diffusivity, which is proportional to the thermal conductivity contribution, versus the slope in relaxation time, normalized by 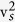. Thus, if both diffusivity and relaxation time are increasing or decreasing together for a group of modes in a certain frequency range, the ratio is positive, despite whether the changes occur at the same rate. However, if the ratio is negative, it implies that some modes have increasing diffusivity, yet decreasing relaxation time, which is fundamentally disjoint with the PGM. Similarly, if the slope in *σ* approaches zero for a group of modes it is because the diffusivity is nearly constant, since Fig. 2c and d show that the relaxation times in both systems are predominantly decreasing with increased temperature. As a result, if the PGM is valid in amorphous materials, then 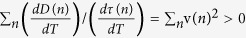 and *σ*(*n*) should always be positive. However, if this ratio is negative, the PGM is inapplicable to amorphous materials in general or at the very least inapplicable to the two materials considered herein.

To test this, we first calculated *σ* for crystalline silicon at three temperatures (100 K, 300 K, 1000 K) using GKMA as shown in Fig. 3(a) and (b). As expected, in both temperature ranges, *σ* is always positive and is roughly constant when comparing the data from different temperature ranges, which suggests that the relaxation time is an appropriate descriptor of the thermal conductivity contributions as determined by the GKMA method, and the PGM is valid. The results in Fig. 3(a) and (b) therefore demonstrate that the proposed approach is useful, since it is consistent with the well-known understanding that the PGM is valid for crystals. For a-Si, on the other hand (see Fig. 3(c)), the modes exhibit positive *σ* when comparing 100–300 K, which implies the relaxation time could be a suitable descriptor in that temperature regime, but Fig. 3(d) shows that between 300–800 K there is a marked change in the behavior and *σ* becomes negative for most modes. This is also apparent from the fact that in Fig. 2(a), the 800K-accumulation curve is higher than at 300 K, yet the relaxation times generally decrease (see Fig. 2(c)).

For a-SiO_2_, as temperature increases, the anharmonicity increases and relaxation times decrease, but thermal conductivity contributions for non-propagating modes (diffusons and locons) do not necessarily decrease with relaxation time. Figure 4 shows *σ* for a-SiO_2_, and interestingly, similar to Fig. 3(c) and (d), *σ* is positive at low temperatures (below 400 K) and starts to decrease with temperature and eventually becomes negative in Fig. 4(d). Thus, one could possibly make the argument that the PGM is still somewhat valid below room temperature, but it becomes questionable when temperature of the system is higher.

## Conclusion

We have conducted a detailed analysis to assess the validity of the PGM. We used lattice dynamics derived phonon frequencies and MD derived temperature dependent relaxation times along with experimentally measured thermal conductivities to back-calculate the phonon velocities for individual modes using the PGM. For many of mid and high frequency modes, the phonon velocity must be either imaginary or unrealistically high (>100X the speed of sound), which is not physical. We therefore concluded that the PGM is not applicable to amorphous solids or at least not a-Si and a-SiO_2_ above room temperature. Using the recently developed GKMA method we calculated the mode diffusivities with inclusion of anharmonicity. Comparing the temperature dependence of mode the diffusivities and relaxation times shows that there is little if any connection between phonon relaxation times and thermal conductivity for the amorphous materials considered herein. Thus, the results provide quantitative evidence that relaxation time is an improper descriptor for the behavior at elevated temperatures in a-Si and a-SiO_2_ and is therefore questionable altogether for amorphous materials.

## Additional Information

**How to cite this article**: Lv, W. and Henry, A. Examining the Validity of the Phonon Gas Model in Amorphous Materials. *Sci. Rep.* **6**, 37675; doi: 10.1038/srep37675 (2016).

**Publisher's note:** Springer Nature remains neutral with regard to jurisdictional claims in published maps and institutional affiliations.

## Supplementary Material

Supplementary Information

## Figures and Tables

**Figure 1:**
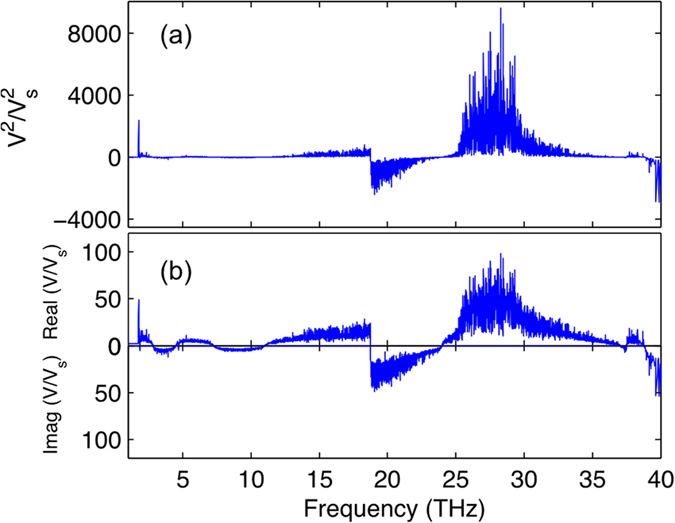
(**a**) Normalized mode phonon velocities squared, (**b**) normalized mode phonon velocities that are real and imaginary for aSiO_2_ calculated from PGM model.

**Figure 2:**
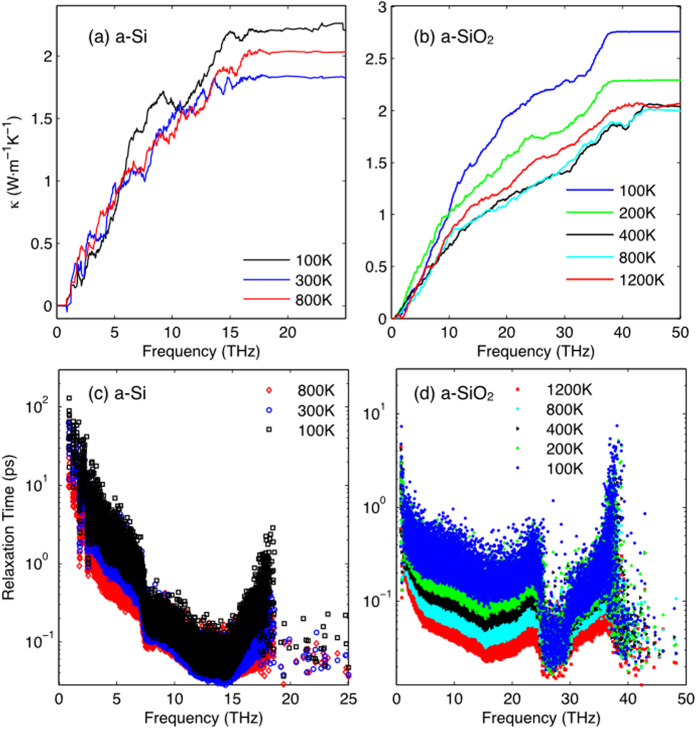
Thermal conductivity accumulation and relaxation times calculated from GKMA. Thermal conductivity accumulation for (**a**) a-Si and (**b**) a-SiO_2_, and relaxation times for (**c**) a-Si and (**d**) a-SiO_2_.

**Figure 3:**
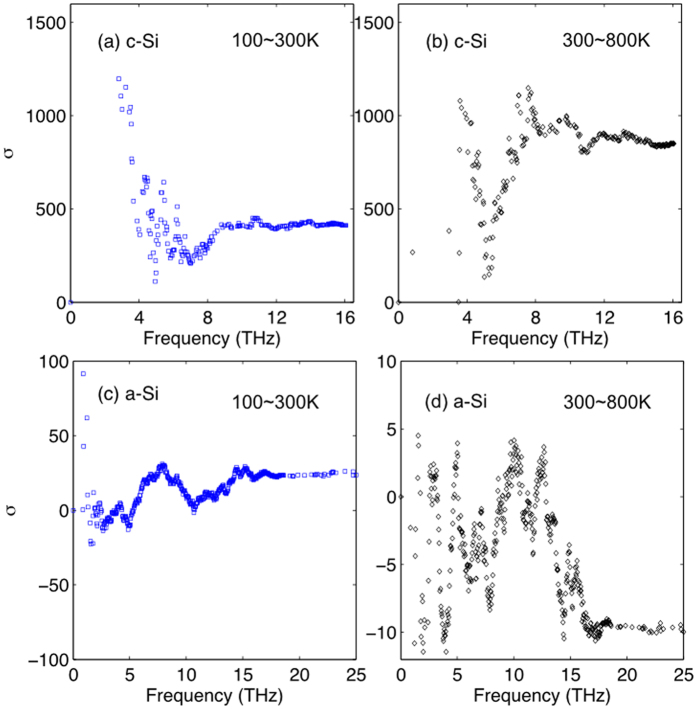
The *σ*(*n*) ratio. (**a**) 100~300 K and (**b**) 300~800 K for c-Si and (**c**) 100~300 K and (**d**) 300~800 K for a-Si.

**Figure 4:**
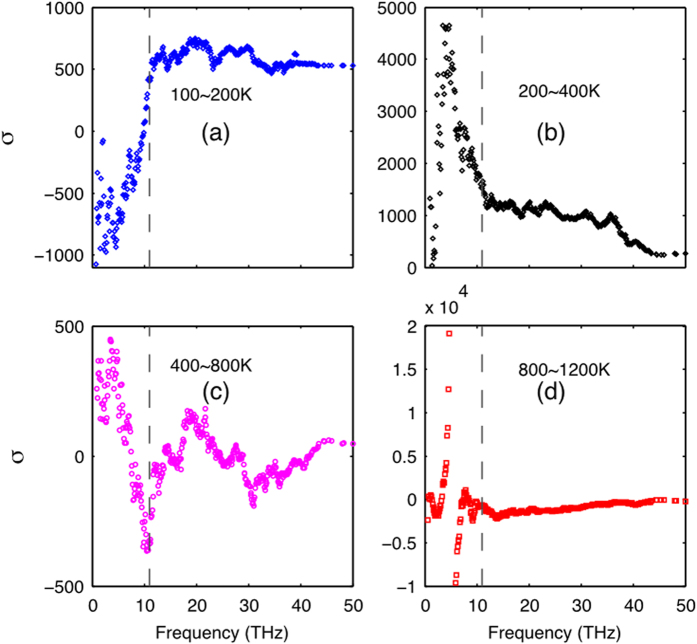
The *σ*(*n*) ratio. (**a**) 100–200 K, (**b**) 200–400 K, **(c)** 400–800 K, and (**d**) 800–1200 K for a-SiO_2_. The dashed curve is the estimated cut-off where the ratio of 
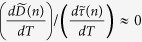
, which is primarily caused by the numerator being close to zero (e.g., constant mode diffusivity).
