# Supplementary material for: Examining the Validity of the Phonon Gas Model in Amorphous Materials
Source: Sci Rep. 2016 Dec 5;6:37675. doi: 10.1038/srep37675 (PMC5137137; doi:10.1038/srep37675)
Supplement: Supplementary Information [file srep37675-s1.pdf]

Examining the Validity of the Phonon Gas Model in Amorphous Materials

Wei Lv[1], Asegun Henry[*, 1,2]

Affiliations:

[1]George W. Woodruff School of Mechanical Engineering, Georgia Institute of Technology, Atlanta, GA 30332, USA.

[2]School of Materials Science and Engineering, Georgia Institute of Technology, Atlanta, GA, 30332, USA.

[*]Corresponding author: ase@gatech.edu

Simulation details of generating a-Si structure

A 4096 atom WWW-generated a-Si structure [1] was used in the calculation with the Tersoff potential[2]. The density of a-Si in the simulation was 2.33 g/cm^3, which is equivalent to the crystalline silicon. How to generate a-Si structure is discussed in references [3,4]. In order to avoid structural meta-stability [4], the initial structures were annealed at 1100K for 10 ns . The simulation time step was 0.25 fs and after 500 ps equilibration with NVT (constant number of atoms, volume and temperature), the modal heat fluxes were written every 1 fs for 6 ns at equilibrium in the microcanonical ensemble at 300K.

Simulation details of generating a-SiO_2 structure

The a-SiO_2 initial structure was generated by the melting-quenching method using Tersoff potential with parameters published by Munetoh et al.[2]. The detailed procedures of melting-quenching method have been described by Ong *et al.*[5]After quenching, the structure was annealed at 1100 K for 10 ns to avoid the meta-stability reported by Larkin *et al*.[4]. After generating the a-SiO_2 structure, we calculated the density of states (DOS), and compared with experiments[6]. The agreement is overall reasonable. The supercell is approximately cubic has 4608 atoms, with a length of ~ 40.44 A. The density for the relaxed structure is 2317 Kg/m^3, which is 4% larger than the experimental value 2220 kg/m^3[7]. The time step used in the simulation is 0.1 fs.

Lattice dynamics simulation and GKMA

After we obtained the initial atomistic structures, we applied lattice dynamics (LD) at gamma point ($k = 0$) for the supercell with periodic boundary conditions to obtain the normal mode eigen values and eigen vectors which allow one to visualize the normal mode shapes. The LD calculations were performed using the General Utility Lattice Program (GULP)[8]. Before LD calculation, we relaxed structure at 0K with zero pressure. Then one obtained the eigenvectors and harmonic frequencies.

Once all the eigen-vectors have been calculated, they are read into the molecular dynamics (MD) simulation to calculate the mode level thermal conductivity contributions from GKMA. All MD simulations were performed using the Large Atomic/Molecular Massively Parallel Simulator (LAMMPS). After equilibrated for 100 ps at 300K using

NVT (constant number of atoms, volume, temperature), the heat flux, and mode heat flux were captured for another 4 ns (4 x 10^7 time steps) using NVE (constant number of atoms, constant volume, constant energy). After supplying the eigenvectors once at the beginning of the MD simulation, one is able to obtain the heat flux and kinetic energy of each mode. The integral of the heat flux autocorrelation function is cut off at 30 ps[9] since the largest relaxation time in the supercell is less than 10 ps. After the modal heat flux is computed, the modal thermal conductivity is determined by calculating the correlation between modal heat flux and the total heat flux.

GKMA formulations

Here, we recapitulate the key points of the GKMA formalism. Firstly, the normal mode eigenvectors are computed from a LD calculation. Secondly, one needs to project the atomic velocities from the MD simulation of the same supercell onto the normal mode eigenvectors. Then one can obtain the time history of each normal mode's amplitude. Thirdly, each atom's instantaneous velocity can then be decomposed into individual mode contributions based on the respective instantaneous normal mode amplitudes, whereby summing the modal contributions returns each atom's velocity. Fourthly, one substitutes the modal components of each atom's velocity into the heat flux operator[10] to obtain each mode's instantaneous contribution to the heat flux. The total heat flux can then be obtained from the sum of all individual mode contributions to the heat flux, via

$$\mathbf{Q}(t)=\sum_{n}^{3N}\mathbf{Q}(n,t)=\sum_{n}^{3N}\frac{1}{V}\sum_{i}\left[E_i\dot{\mathbf{x}}_i(n,t)+\sum_{j}(-\nabla_{\mathbf{r}_i}\Phi_j\cdot\dot{\mathbf{x}}_i(n,t))\mathbf{r}_{ij}\right] \quad (1)$$

where n is mode index, N is total number of atoms in the super cell, V is volume of the super cell, E_i is the kinetic and potential energy of atom i, Φ_j denotes potential energy of atom j, $\dot{\mathbf{x}}_i(n,t)$ is the contribution mode n makes to the velocity of atom i and $\mathbf{r}_{ij}$ is distance between atom i and j. After having access to the individual mode heat fluxes, we can substitute the summation over modes in Eq. (1) directly into the Green-Kubo expression for TC, which calculates the TC as proportional to the heat flux autocorrelation function. One then obtains the TC as a direct summation over individual mode contributions,

$$\kappa=\sum_{\mathrm{n}}\kappa(\mathrm{n})=\sum_{\mathrm{n}}\frac{\mathrm{V}}{\mathrm{k_B T^2}}\int_0^{\infty}\langle\mathbf{Q}(\mathrm{n,t})\cdot\mathbf{Q}(0)\rangle\mathrm{dt} \quad (2)$$

where $\kappa(\mathrm{n})$ stands for TC contribution of mode n, $\mathrm{k_B}$ is Boltzmann constant, T is the temperature and V is volume. Using Eq. (2) one can calculate the TC of individual modes in any material where the atoms vibrate around stable equilibrium sites. Classical MD has been considered to be inaccurate at low temperatures (i.e., below a material's Debye temperature) because it does not reproduce the proper mode amplitudes that correspond to the quantum occupations. As a result, classical MD results in a constant heat capacity with respect to temperature, since every mode is equally excited at all temperatures. However, once each individual mode's TC is obtained, one can easily apply a quantum specific heat correction, which allows one to extend the MD based predictions to essentially any temperature.

Quantum specific heat correction

The quantum expression of volumetric specific heat, based on Bose-Einstein statistics is given by [14],

$$c_q(\omega) = \frac{k_B x^2}{V}\frac{\exp(x)}{[\exp(x)-1]^2}; x = \frac{\hbar\omega}{k_B T} \quad (3)$$

and the classical volumetric specific heat is given by $c_m(\omega) = \frac{k_B}{V}$. Thus, the quantum heat capacity correction factor is the ratio,

$$c_q(\omega)/c_m(\omega) = \frac{x^2\exp(x)}{[\exp(x)-1]^2} \quad (4)$$

Calculation of phonon group velocities using PGM

The relaxation time $\tau(n)$ is calculated using NMA method with Tersoff potential on a-SiO_2 at six different temperatures (30K, 100K, 100K, 200K, 400K, 800K). Then one could easily calculate relaxation time $\tau(n,T)$ ($30K \le T \le 800K$) by interpolating between different temperatures. The specific heat from Bose-Einstein statistics [14] is also temperature dependent, as $c(n,T) = \frac{k_B \exp(\hbar\omega(n)/k_B T)}{V\left[\exp(\hbar\omega(n)/k_B T)-1\right]^2}$. Assume group velocity $v_g(n)$ is independent of temperature. The measured thermal conductivity of a-SiO_2 is available from 30K to 750K [2]. The first step is to get temperature dependent thermal conductivity as a continuous function $\kappa(T)$ by fitting from the experiments result. Then start from 30K, we could calculate the square of phonon group velocity $v_g(n)^2$ from $v_g(n)^2 = \kappa(T)/\left[c(T,n)\tau(T,n)\right]$. At 30K, there are only a few modes are excited, in other words, most of modes $c(n,30)$ is negligible. We choose $c(n,T) < 0.001$ as a criteria to set mode n is excited or not at temperature T. After we calculated the phonon velocities of a few modes that are excited at 30K, we increased temperature to be slightly higher (30.072K). Since there are total 13824 modes in the system, we divided temperature into 10000 bins so that each temperature increase only excites a small number of modes. When the temperature changes, the relaxation time and specific heat are changing for all modes. The contribution of thermal conductivity $\sum_n^{n'} k(n,T)$ from the modes that phonon velocity has been calculated is changing with the relaxation time and specific heat. The difference between experimental value $k(T)$ and $\sum_n^{n'} k(n,T)$ is used to determine the phonon velocity of newly excited modes at this temperature using $v_g(n)^2 = \left[\kappa(T) - \sum_n^{n'} k(n,T)\right]/\left[c(T,n)\tau(T,n)\right]$. Iteratively, we calculated the square of phonon velocities for all modes.

References:

1. Barkema, G. T. & Mousseau, N. High-quality continuous random networks. *Phys. Rev. B* **62,** 4985 (2000).
2. Munetoh, S., Motooka, T., Moriguchi, K. & Shintani, A. Interatomic potential for Si--O systems using Tersoff parameterization. *Comput. Mater. Sci.* **39,** 334–339 (2007).
3. He, Y., Donadio, D. & Galli, G. Heat transport in amorphous silicon: Interplay between morphology and disorder. *Appl. Phys. Lett.* **98,** 144101 (2011).
4. Larkin, J. M. & McGaughey, A. J. H. Thermal conductivity accumulation in amorphous silica and amorphous silicon. *Phys. Rev. B* **89,** 144303 (2014).
5. Ong, Z.-Y. & Pop, E. Molecular dynamics simulation of thermal boundary conductance between carbon nanotubes and SiO_{2}. *Phys. Rev. B* **81,** 155408 (2010).
6. Carpenter, J. & Price, D. Correlated Motions in Glasses Studied by Coherent Inelastic Neutron Scattering. *Phys. Rev. Lett.* **54,** 441–443 (1985).
7. Kaviany, M. *Principles of Heat Transfer*. (John Wiley & Sons, 2002).
8. Gale, J. D. GULP: A computer program for the symmetry-adapted simulation of solids. *J. Chem. Soc. Faraday Trans.* **93,** 629–637 (1997).
9. McGaughey, A. J. H. & Kaviany, M. Thermal conductivity decomposition and analysis using molecular dynamics simulations: Part II. Complex silica structures. *Int. J. Heat Mass Transf.* **47,** 1799–1816 (2004).
10. Lv, W. & Henry, A. Direct calculation of modal contributions to thermal conductivity via Green–Kubo modal analysis. *New J. Phys.* **18,** 13028 (2016).
11. Henry, A. S. & Chen, G. Spectral Phonon Transport Properties of Silicon Based on Molecular Dynamics Simulations and Lattice Dynamics. *Journal of Computational and Theoretical Nanoscience* **5,** 141–152 (2008).
12. Henry, A. & Chen, G. High Thermal Conductivity of Single Polyethylene Chains Using Molecular Dynamics Simulations. *Phys. Rev. Lett.* **101,** 235502 (2008).
13. van Duin, A. C. T., Dasgupta, S., Lorant, F. & Goddard, W. A. ReaxFF: A Reactive Force Field for Hydrocarbons. *J. Phys. Chem. A* **105,** 9396–9409 (2001).
14. Ziman, J. M. *Electrons and phonons: the theory of transport phenomena in solids*. *Endeavour* **20,** (1960).
15. Cahill, D. G. Thermal conductivity measurement from 30 to 750 K: the 3ω method. *Rev. Sci. Instrum.* **61,** 802 (1990).
